# Previously Unrecognized Ornithuromorph Bird Diversity in the Early Cretaceous Changma Basin, Gansu Province, Northwestern China

**DOI:** 10.1371/journal.pone.0077693

**Published:** 2013-10-11

**Authors:** Ya-Ming Wang, Jingmai K. O'Connor, Da-Qing Li, Hai-Lu You

**Affiliations:** 1 Institute of Geology, Chinese Academy of Geological Sciences, Beijing, People’s Republic of China; 2 Key Laboratory of Vertebrate Evolution and Human Origins of Chinese Academy of Sciences, Beijing, People’s Republic of China; 3 Institute of Vertebrate Paleontology and Paleoanthropology, Chinese Academy of Sciences, Beijing, People’s Republic of China; 4 Gansu Geological Museum, Chengguan District, Lanzhou, Gansu Province, People’s Republic of China; Raymond M. Alf Museum of Paleontology, United States of America

## Abstract

Here we report on three new species of ornithuromorph birds from the Lower Cretaceous Xiagou Formation in the Changma Basin of Gansu Province, northwestern China: *Yumenornis huangi* gen. et sp. nov., *Changmaornis houi* gen. et sp. nov., and *Jiuquanornis niui* gen. et sp. nov.. The last of these is based on a previously published but unnamed specimen: GSGM-05-CM-021. Although incomplete, the specimens can be clearly distinguished from each other and from *Gansus yumenensis* Hou and Liu, 1984. Phylogenetic analysis resolves the three new taxa as basal ornithuromorphs. This study reveals previously unrecognized ornithuromorph diversity in the Changma avifauna, which is largely dominated by *Gansus* but with at least three other ornithuromorphs. Body mass estimates demonstrate that enantiornithines were much smaller than ornithuromorphs in the Changma avifauna. In addition, Changma enantiornithines preserve long and recurved pedal unguals, suggesting an arboreal lifestyle; in contrast, Changma ornithuromorphs tend to show terrestrial or even aquatic adaptions. Similar differences in body mass and ecology are also observed in the Jehol avifauna in northeastern China, suggesting niche partitioning between these two clades developed early in their evolutionary history.

## Introduction

In the last decade, nearly one hundred fossil birds have been discovered from the Lower Cretaceous Xiagou Formation in the Changma Basin of Gansu Province in northwestern China. Most of these are referred to *Gansus yumenensis*, the first Mesozoic fossil bird from China [1-4], although additional diversity has slowly accumulated; a second unnamed ornithuromorph bird [5], the enantiornithine *Qiliania graffini* [6], and three other unnamed enantiornithine specimens [7-9] have also been described. 

The second unnamed ornithuromorph specimen, GSGM-05-CM-021, consists of an isolated sternum, furcula, and sternal ribs [5]. Its sternum is distinct from that of *Gansus*, representing a basal non-ornithurine member of Ornithuromorpha similar to *Archaeorhynchus* from the Jiufotang Formation of Liaoning Province in northeastern China [5,10]. Despite this growing diversity, all other taxa are represented by isolated specimens and *Gansus* is by far the dominant taxon in the Changma avifauna.

Here we describe two new ornithuromorph specimens from the Xiagou Formation in the Changma Basin. Based on comparison with GSGM-05-CM-021 [5,10], we also erect a new species for this specimen. We describe the morphology of the new specimens and discuss the significance of these taxa in understanding the Changma avifauna.

## Methods

Anatomical nomenclature primarily follows Baumel and Witmer [11]. English equivalents are used for osteological terminology while Latin is maintained for muscles. All measurements were taken with calipers to the nearest 0.1 mm. 

The two new specimens, GSGM-06-CM-013 and GSGM-08-CM-002, and the previously described GSGM-05-CM-021 (Gansu Geological Museum) were discovered in the Xiagou Formation near Changma in Gansu Province of northwestern China. Precise locality information is available to qualified researchers upon request. The issuing authority is the Gansu Geological Museum, represented by Da-Qing Li (co-author). All necessary permits were obtained for the described study, which complied with all relevant regulations.

A phylogenetic analysis was performed using the O’Connor and Zhou [12] dataset, with the addition of *Yumenornis*, *Changmaornis*, and *Jiuquanornis* [5] (File S1). Neornithes was represented by *Anas platyrhynchos* and *Gallus gallus*; Dromaeosauridae was scored as the outgroup. The dataset consists of 245 characters (31 ordered; all characters weighted equally) evaluated for 63 taxa; the matrix was analyzed with TNT [13], applying a heuristic search retaining the single shortest tree from every 1000, followed by an additional round of tree bisection reconnection (TBR) branch swapping.

### Nomenclatural Acts

The electronic edition of this article conforms to the requirements of the amended International Code of Zoological Nomenclature, and hence the new names contained herein are available under that Code from the electronic edition of this article. This published work and the nomenclatural acts it contains have been registered in ZooBank, the online registration system for the ICZN. The ZooBank LSIDs (Life Science Identifiers) can be resolved and the associated information viewed through any standard web browser by appending the LSID to the prefix "http://zoobank.org/". The LSID for this publication is: urn:lsid:zoobank.org:pub:DC6A3D74-B8B7-41BA-A6BF-7BD48D6CD3C7. The electronic edition of this work was published in a journal with an ISSN, and has been archived and is available from the following digital repositories: PubMed Central, LOCKSS.

## Results

### Locality and Horizon

**Figure 1:**
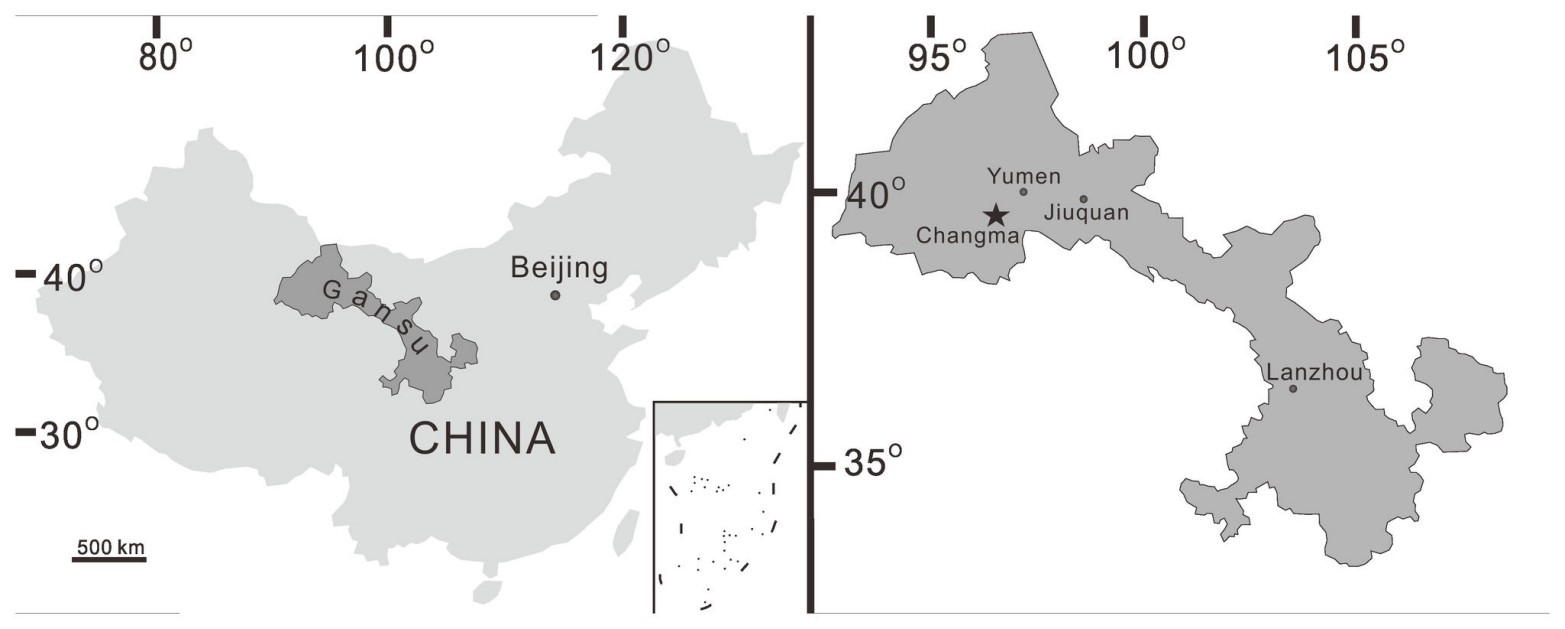
Geographic location of the Changma Basin (indicated by star), Gansu Province, People’s Republic of China.

In the vicinity of the Changma Township, Yumen City, Jiuquan area, northwestern Gansu Province, China; Lower Cretaceous (early Aptian), Xiagou Formation [7-9,14] (Figure 1).

### Systematic Paleontology

Aves Linnaeus, 1758 [15]
Pygostylia Chiappe, 2002 [16]
Ornithothoraces Chiappe, 1995 [17]
Ornithuromorpha Chiappe, 2002[16]
*Yumenornis* *huangi* gen. et sp. nov.

urn:lsid:zoobank.org:act:EEC6E8A1-318F-491B-BD62-EFF58C4DBFC0

### Holotype

**Figure 2:**
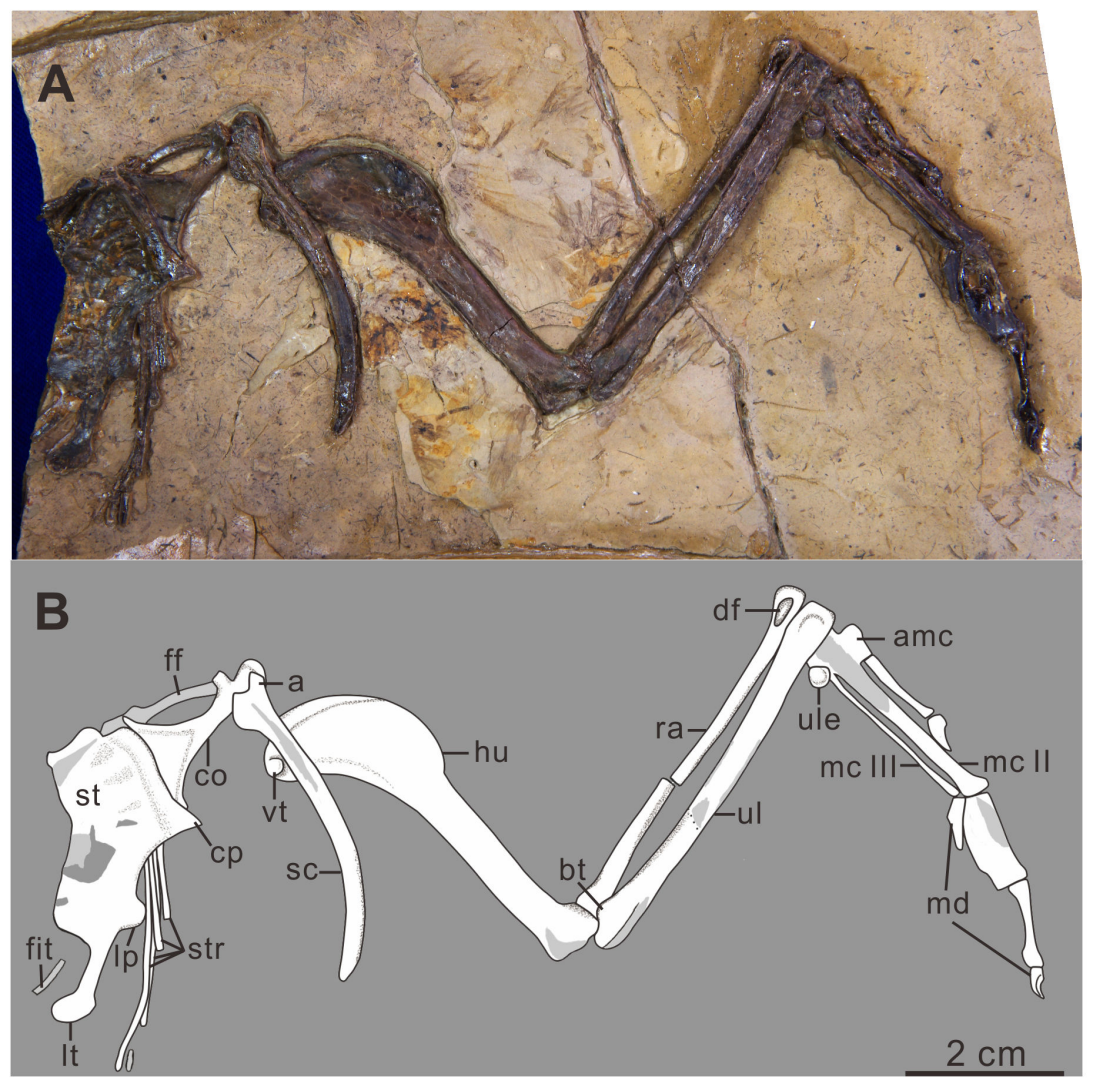
Photograph of *Yumenornis huangi* gen. et sp. nov., GSGM-06-CM-013 (A) and line drawing (B). Abbreviations: a, acromion; amc, alular metacarpal; bt, bicipital tubercle; co, coracoid; cp, craniolateral process; df, distal fossa; ff, fragment of the furcula; fit, fragment of the intermediate trabecula; hu, humerus; lp, lateral process; lt, lateral trabecula; mc II, metacarpal II; mc III, metacarpal III; md, manual digits; sc, scapula; st, sternum; str, sternal ribs; ra, radius; ul, ulna; ule, ulnare; vt, ventral tubercle.

Gansu Geological Museum (GSGM)-06-CM-013, partial sternum, complete right coracoid and scapula, partial furcula, and a complete, articulated right forelimb (Figure 2). 

### Etymology

The generic name “Yumen” is derived from the name of the city near the fossil locality. The specific name is dedicated to Mr. Zhao-Chu Huang of the Institute of Vertebrate Paleontology and Paleoanthropology (IVPP) for his long-lasting support to several generations of IVPP staff. 

### Diagnosis

*Yumenornis huangi* is distinguished from other known ornithuromorphs by the following unique combination of characters: sternum with angular rostral margin (~90°), lateral (zyphoid) processes, and robust, distally expanded lateral trabeculae; radius with deep distal fossa; ratio of length of manus relative to humerus 1.1. *Yumenornis huangi* can be separated from *Gansus* by the following morphological differences: the rostral margin of the sternum is sharper than that of *Gansus* (90° vs. 140° in *Gansus*); the lateral trabeculae are short and caudally expanded, contrasting with the unexpanded trabeculae in *Gansus*. 

### Description

**Figure 3:**
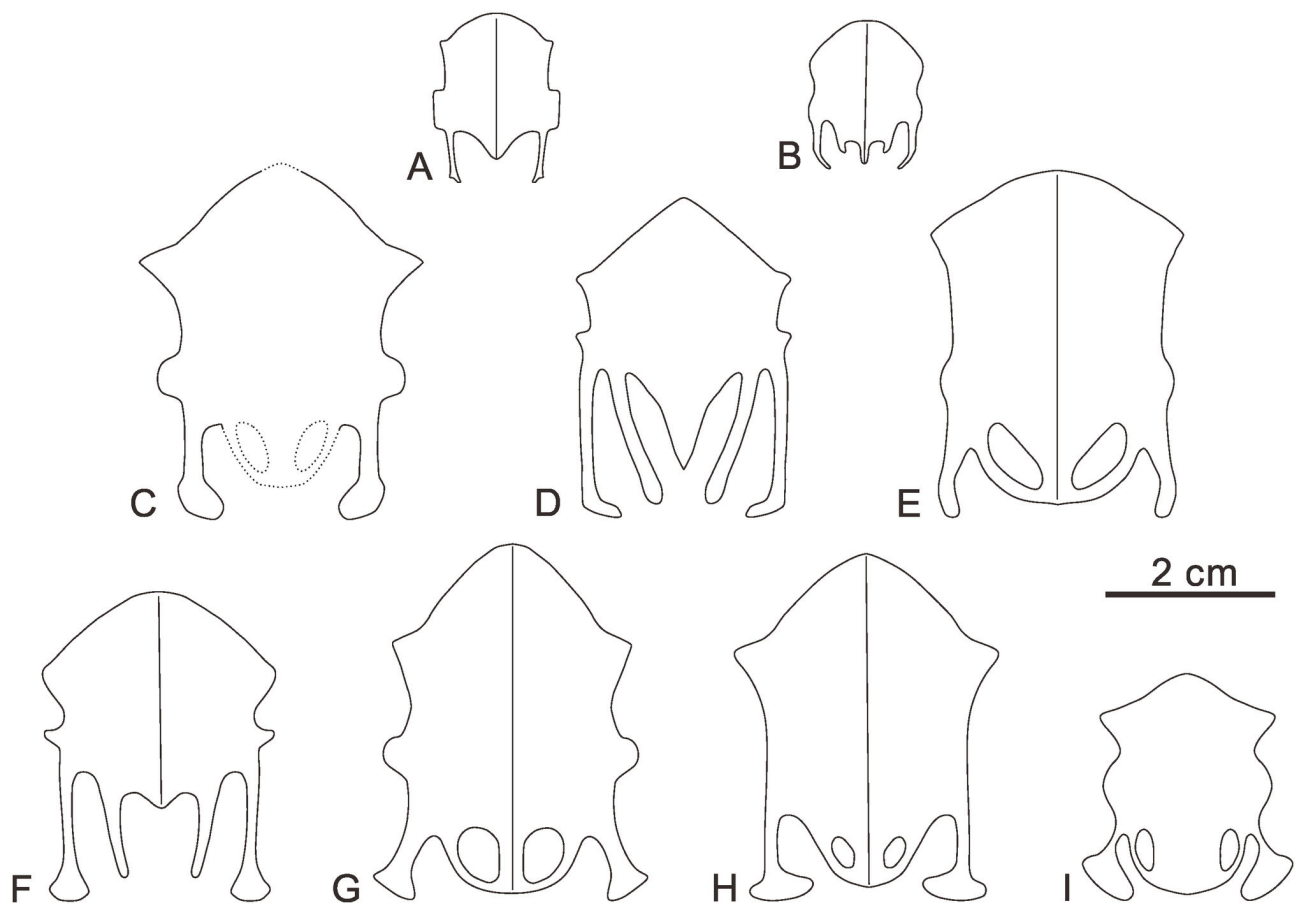
Comparison of sterna of *Yumenornis huangi* (C) and *Jiuquanornis niui* (D) with other Early Cretaceous ornithuromorphs. A, *Longicrusavis houi*; B, *Hongshanornis longicresta*; E, *Gansus yumenensis*; F, *Archaeorhynchus spathula*; G, *Yixianornis grabaui*; H, *Yanornis martini*; I, *Songlingornis linghensis*. Dashed lines in C represent estimated reconstruction.

The sternum is preserved in dorsal view, lacking the left half (Figure 2). The rostral margin defines an angle of approximately 90°, similar to *Jianchangornis microdonta*, *Yanornis martini* (Figure 3H), and the unnamed ornithuromorph GSGM-05-CM-021 (*Jiuquanornis niui*) (Figure 3D) also from Changma [5,18,19], but contrasting with the greater angle in *Gansus* (Figure 3E) (approximately 140°; GSGM-06-CM-011). A small notch is present on the midline of the rostral margin, indicating the coracoids were separated mediolaterally; but this notch may be due to preservation. The coracoidal sulci are well developed as relatively deep cranial grooves. The dorsal surface is concave, as in most flying neornithines. The right craniolateral process, although possibly flattened during diagenesis, projects craniolaterally, and defines a sharp angle of nearly 65°, similar to that of *Yixianornis grabaui* (Figure 3G), *Yanornis martini* [19,20], *Songlingornis linghensis* (Figure 3I) [12,21], and GSGM-05-CM-021 [5]. In *Gansus* (GSGM-06-CM-011) the craniolateral process defines a wider angle of approximately 100° and situates more cranially than that of *Yumenornis*. Four sternal ribs are preserved in articulation with the lateral margin, which is less than those in *Gansus* (five costal facets; GSGM-04-CM-020) and GSGM-05-CM-021 (potentially five costal facets) [5]. A rounded lateral (zyphoid) process is present prominently on the lateral margin, close to the cranial end of the lateral trabecula, similar to that of *Yixianornis* [20]. The lateral processes in *Songlingornis* and *Gansus* are more quadrangular, while in GSGM-05-CM-021 they are small and triangular. The lateral trabecula is short and robust, with a large fan-shaped caudal expansion, similar to that in *Yixianornis*, *Yanornis*, and *Songlingornis*; in *Gansus*, the lateral trabeculae are shorter, thinner, and slightly curved medially, without a caudal expansion (Figure 3E). The intermediate trabecula is damaged, with only a fragment remaining; it is thinner than the lateral trabecula and appears to curve medially, similar to *Ambiortus dementjevi* [22], *Gansus*, *Songlingornis*, *Yanornis*, and *Yixianornis*. Whether this process contacted the xiphial region defining a fenestra, as in *Gansus*, *Songlingornis*, *Yanornis*, and *Yixianornis*, cannot be determined. The preserved morphology of the sternum of *Yumenornis* is most similar to that of *Yixianornis* and *Songlingornis*; all three taxa have sharp craniolateral processes, large and rounded lateral processes, and caudal expansions of the lateral trabeculae.

The right coracoid is strut-like, preserved in dorsal view; however, it is partially covered proximally by the scapula and distally by the sternum (Figure 2). The rounded acrocoracoid process appears to be hooked medially, although it is somewhat destroyed. The procoracoid process is well-developed and projected medially. The body of the coracoid bears a large, shallow, and triangular fossa for the attachment of the m. sternocoracoidei impression, as in other Early Cretaceous ornithuromorphs (e.g. *Gansus*, *Yanornis*, and *Jianchangornis*). The medial and lateral margins are approximately the same length, and are both strongly concave, forming an expanded sternal margin, as in *Yanornis* and *Yixianornis*. In *Gansus*, the lateral margin is more strongly concave than the medial margin. The sternal margin is covered by the sternum, and the presence of a lateral process, like that present in most ornithuromorphs, cannot be determined. The right scapula is completely preserved but slightly crushed proximally, obscuring most details of the articular surfaces. The corpus is curved and slightly tapered distally. The acromion process is short, only extending slightly beyond the coracoidal tubercle, contrasting with the longer process in *Gansus*. A delicate, curved fragment of bone is interpreted as part of the right furcular ramus. 

**Figure 4:**
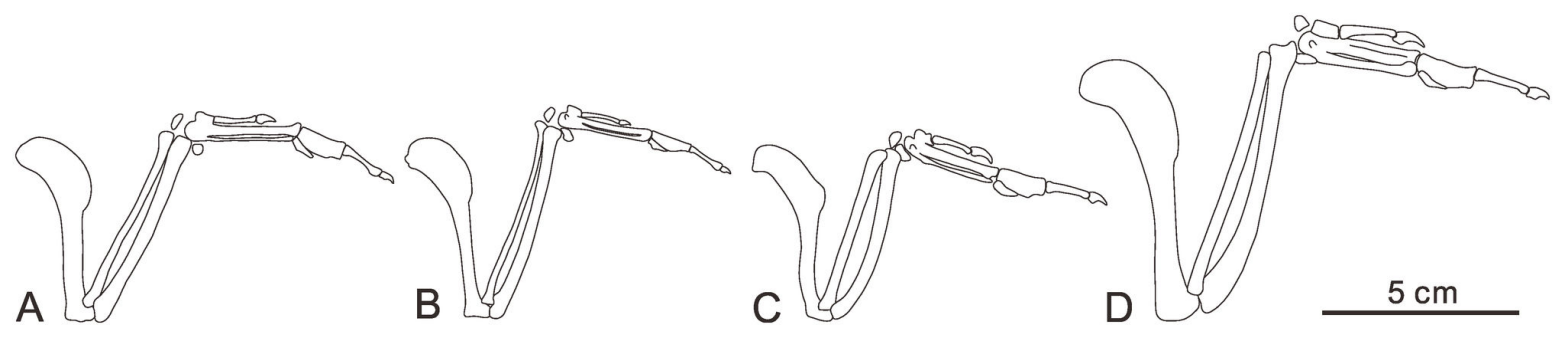
Comparison of forelimbs of *Yumenornis huangi* (A) and other Early Cretaceous ornithuromorphs. B, *Gansus yumenensis*; C, *Yixianornis grabaui*; D, *Yanornis martini*.

The right humerus is completely preserved in caudal view, although covered by the scapula proximoventrally and badly crushed distally (Figure 2). The humeral head appears globose: it is proximally and caudally convex, and projects more proximally than the deltopectoral crest, like most ornithuromorphs. The small ventral tubercle is weakly separated from the humeral head by a shallow capital incision; it is weakly excavated ventrally by a shallow pneumotricipital fossa. The deltopectoral crest extends for 44% the total length of the humerus, similar to that of *Gansus* (43%, GSGM-05-CM-014); the width of the crest is slightly less than that of the shaft (wider than in *Yanornis* but narrower than in *Gansus* and hongshanornithids) [23]. The crest tapers into the shaft distally, as in most Early Cretaceous ornithuromorphs. The ratio of the width of the midshaft to the total length of humerus in *Yumenornis* is 0.1 (Figure 4A), much larger than those observed in specimens of *Gansus* GSGM-05-CM-014 (0.07) and GSGM-IG-04-CM-004 (0.06) (Figure 4B); the robust humerus resembles those of *Yanornis* (0.1, IVPP V13278) and *Yixianornis* (0.1, IVPP V13631) from The Jehol Group (Figure 4C, D). 

The ulna and radius are both severely damaged (Figure 2). The ulna is longer than the humerus and only slightly bowed, similar to the condition in *Gansus*, but unlike the relatively short and strongly bowed ulnae of *Yixianornis* and *Yanornis* (Figure 4). Proximally, the olecranon process is short and rounded. The bicipital tubercle is larger and more rounded than that of *Gansus*. An extremely deep fossa is present on the lateral surface of the distal end of the radius, nearly perforating the bone; this fossa appears more likely to be formed naturally than the result of preparation. Only the ulnare is preserved; it bears a shallow, round fossa on the possibly dorsal ramus, resembling that of *Gansus*. Both the ulna and radius are more robust than those in *Gansus* (Table 1), while similar to those of *Yixianornis*, respectively (IVPP V13631; Figure 4) [20].

**Table 1:** Measurements (mm) of forelimbs of *Gansus yumenensis* (CAGS-IG-04-CM-004, GSGM-05-CM-014) and *Yumenornis huangi* gen. et sp. nov. (GSGM-06-CM-013).

Measurement	Specimen		
	CAGS-IG-04-CM-004	GSGM-05-CM-014	GSGM-06-CM-013
Humerus length	48.0	47.8	49.9
Ulna length	49.1	51.1	52.9
Ulna midshaft width	2.9	3.0	3.6
Radius length	46.7	48.9	49.7
Radius midshaft width	1.6	1.7	2.0
Carpometacarpus length	23.2	23.7	27.0
Metacarpal II width	1.8	1.4	2.0
Metacarpal III width	0.8	0.7	0.9
Phalanx I-1 length	8.8	9.6	10.9
Phalanx I-2 length	3.9	3.6^e^	5.4
Phalanx II-1 length	9.7	—	12.1
Phalanx II-2 length	9.0	—	11.5
Phalanx II-3 length	3.2	2.8	4.2
Phalanx III-1 length	—	—	6.7
Phalanx III-2 length	—	5.8	6.3

*e* Estimated value.

The right carpometacarpus is preserved in dorsal view; the proximal end is heavily damaged and the degree of fusion is unclear (Figure 2). The alular metacarpal is short and nearly rectangular; the cranial half is convex, developed into a small extensor process that only slightly surpasses the distal articular facet for phalanx I-1in cranial extent, similar to those in *Gansus* and *Yixianornis*, but less developed than that of *Apsaravis ukhaana* [24]. Metacarpals II and III are both straight. Metacarpal II is much wider than metacarpal III, which is very thin. Their distal ends appear not to be fused or are only partially fused, suggesting the specimen is a subadult; metacarpal II extends distally slightly farther than metacarpal III, and both the distal ends are expanded. The two metacarpals define a narrow intermetacarpal space that is more than twice the width of metacarpal III (narrower in *Gansus*; Figure 4).

The manual digits are preserved completely in articulation (Figure 2). Phalanx I-1 is slightly bowed and tapered distally, but expanded at the articular surface. Phalanx I-2 is a weakly recurved claw. This digit is very short, ending well proximal to the distal end of metacarpal II, as in other ornithuromorphs. Phalanx II-1 is craniocaudally expanded to nearly twice the width of metacarpal II. Phalanx II-2 is approximately the same length as II-1, but is thin, slightly bowed, and tapered medially so that the proximal and distal ends are both expanded relative to the shaft. Phalanx II-3 is an ungual, smaller than that of the alular digit, bearing a shallow extensor groove along its length. Phalanx III-1 is relatively slender, slightly less than half the length of II-1 and less than one third the width. It is wedge-shaped and slightly tapered distally, with a small, pointed, caudally directed tubercle. All the manual phalanges of *Yumenornis* are proportionately longer and more robust than those of *Gansus* (Table 1), but equal to those of *Yixianornis* (IVPP V 13631; Figure 4) [20]. 

*Changmaornis houi* gen. et sp. nov.

urn:lsid:zoobank.org:act:8E3453BC-A004-4383-BC3C-A889F0F9E2B9

### Holotype

**Figure 5:**
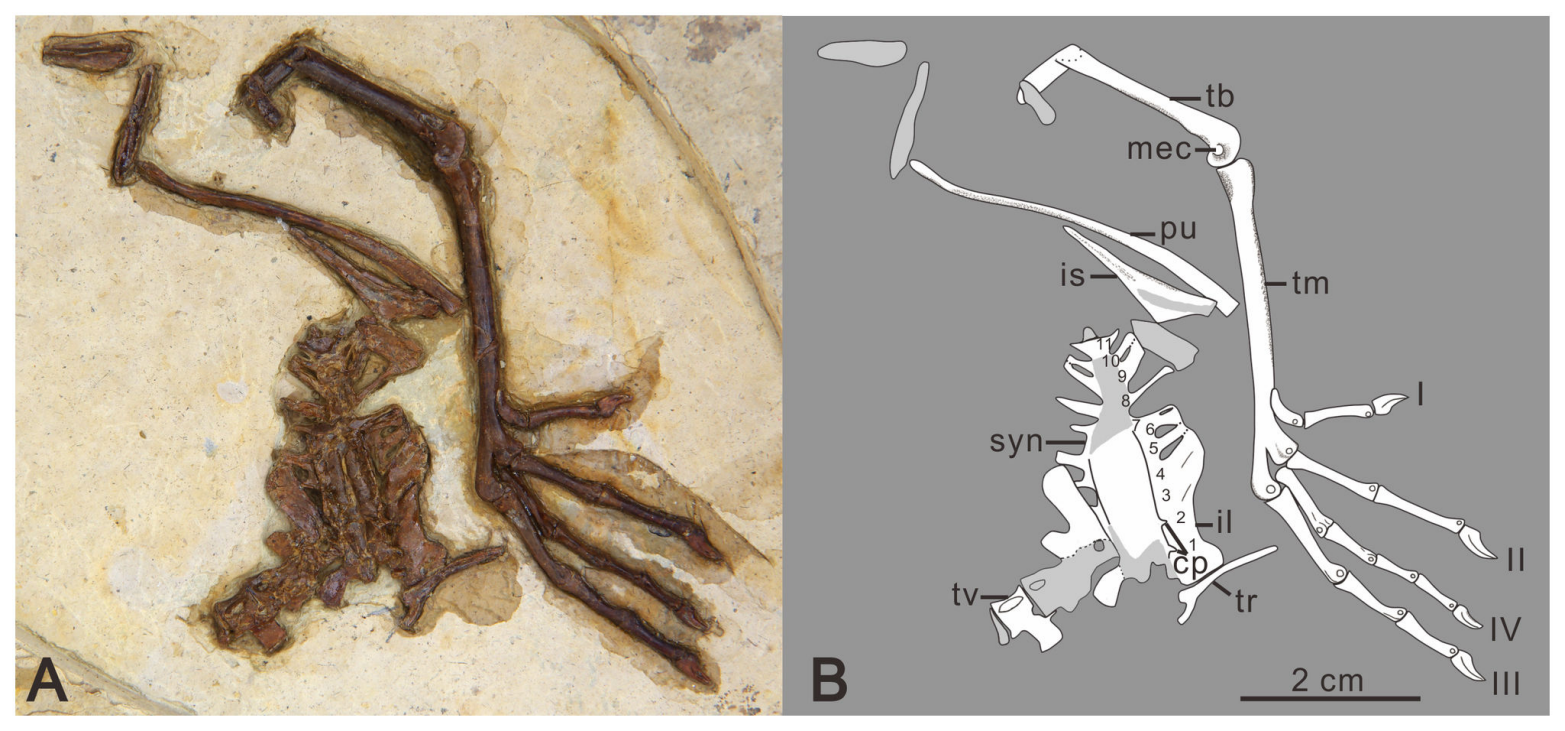
Photograph of *Changmaornis houi* gen. et sp. nov., GSGM-08-CM-002 (A) and line drawing (B). Abbreviations: cp, costal process; il, ilium; is, ischium; mec, medial epicondyle; pu, pubis; syn, synsacrum; tb, tibiotarsus; tm, tarsometatarsus; tr, thoracic rib; tv, thoracic vertebra; I- IV, pedal digit I- IV. Numbers 1-11 in B indicate the number of the sacral vertebrae.

GSGM-08-CM-002, two thoracic vertebrae, a thoracic rib, partial synsacrum, a nearly complete right ischium and pubis, right distal tibiotarsus, and a complete, articulated right foot (Figure 5).

### Etymology

The generic name “Changma” is derived from the name of the town where the fossil locality belongs to. The specific name is in honor of Professor Lian-Hai Hou, who named *Gansus yumenensis*.

### Diagnosis

*Changmaornis houi* is distinguished from other known ornithuromorphs by the following unique combination of characters: synsacrum composed of at least 11 sacral vertebrae with elongate distal transverse processes; ischium with dorsal process; distal half of the pubis compressed mediolaterally; metatarsal I J-shaped; distal margin of metatarsal II trochlea does not reach the proximal margin of the metatarsal III trochlea; pedal digit III longest in foot; ratio of pedal digit III to tibiotarsus 0.82; robust and blunt pedal unguals with poorly developed flexor tubercles. *Changmaornis houi* can be distinguished from *Gansus* by the following morphological differences: pedal digit III is the longest and shorter than tarsometatarsus, while in *Gansus* pedal digit IV is the longest and longer than tarsometatarsus; pedal unguals lack well-developed flexor tubercles, contrasting with the pointed flexor tubercles in *Gansus*.

### Description

A relatively complete thoracic vertebra is preserved in articulation with another completely damaged thoracic (Figure 5). The spinous process is rectangular, with a distinct ridge forming the dorsal margin. The vertebral centrum is laterally excavated by a deep, broad fossa, similar to those of other basal ornithuromorphs (e.g. *Gansus*, *Yixianornis*, *Yanornis*, and *Hongshanornis longicresta* [25]). The length of the centrum is nearly twice its width. The articular surfaces appear to be amphicoelous. The proximal half of an isolated thoracic rib is preserved. It bears a round costal tubercle on the proximal end. The synsacrum is preserved in ventral view, crushed so that the cranial and caudal ends are unclear. The two cranialmost sacral vertebrae bear laterodorsally directed costal processes. The synsacrum is formed by at least 11 completely fused vertebrae based on the number of preserved transverse processes (Figure 5B). In most ornithuromorphs, the synsacrum is composed of at least nine vertebrae; *Yixianornis* and *Yanornis* both have nine fused sacral vertebrae [19,20], while the more derived *Gansus* [2], *Apsaravis* [24], and *Ichthyornis* [26] possess 10 fused sacrals. The basalmost ornithuromorph, *Archaeorhynchus spathula*, possesses only seven to eight fused sacral vertebrae [10,12,27], although this may potentially be ontogenetic given that all known specimens are subadult [10]. In enantiornithines, the synsacrum is composed of seven or eight vertebrae [12,28]. The transverse processes of the sacral vertebrae are much longer than those of *Gansus* [2]; in the first few vertebrae they are laterocaudally directed, becoming more caudally directed and elongate in the last four synsacral vertebrae, similar to *Yanornis* [19]. 

Of the two ilia, only the proximal half of the right side and a fragment of the left are preserved, although these do not provide many anatomical details; the lateral margins appear to be deeply concave in ventral view (Figure 5). The right ischium and pubis are not preserved in articulation and are missing their proximal ends. The dorsal process of the ischium is large and obtusely triangular, as in *Gansus* and other ornithuromorphs, but is more proximally located than in *Yixianornis*. The distal end of the ischium tapers to its terminus, as in *Gansus* and *Yixianornis*. The pubis is medially curved and mediolaterally compressed along its distal half, contrary to the uncompressed pubis in *Gansus*. The distal end of the pubis does not expand ventrally although a dorsal expansion may have been present. 

The distal end of the right tibiotarsus is preserved in medial view, in articulation with the completely preserved right tarsometatarsus and pedal phalanges (Figure 5). A medial epicondyle is developed on the medial surface of the distal tibiotarsus. Two tubercles are developed on the cranial surface of the distal end of the tibiotarsus. One is subrounded, located midway between the medial and lateral condyles, and bounded by a shallow fossa. The other is developed proximal to the medial condyle, forming a cranially projecting, proximodistally elongate ridge. The shape and position of the two tubercles resemble the paired ridges present in *Apsaravis*, which are interpreted as comparable to the tubercles for attachment of the extensor retinaculum in Neornithes [24]. However, three tubercles are present cranially on the distal end of the tibiotarsus in *Gansus* (GSGM-05-CM-014): the distalmost tubercle is subtriangular; the next tubercle is smaller, and located proximal and lateral to the first; the proximalmost one is medially located, forming an elongate ridge similar to the second one just described for *Changmaornis*. 

As in *Gansus*, the tarsometatarsus is straight and completely fused (Figure 5). The dorsal surface of the proximal end is somewhat crushed; a large prominence without grooves or ridges projects weakly on the plantar surface of the proximal end, interpreted as a primitive hypotarsus, similar to that in *Gansus* and other basal ornithuromorphs (e.g. *Yixianornis* and *Yanornis*) [20,29]. The proximodorsal surface bears a deep dorsal infracotylar fossa, and the tubercle for the attachment of the m. tibialis cranialis is located on the mediodistal margin of this fossa [11]. Metatarsal IV is buried in the matrix; only metatarsals I, II, and III are visible. The short metatarsal I is nearly “J”-shaped in medial view, articulating with the medial surface of metatarsal II proximal to the trochlea, similar to the condition in *Gansus*. A small collateral ligament pit is present on the medial surface of the trochlea of metatarsal I. Metatarsal II does not extend distally to the base of the metatarsal III trochlea, and is displaced plantarly; the proximal position of metatarsal II trochlea strongly resembles that in *Gansus*. The trochlear surface of metatarsal II is partially ginglymous, while metatarsal III is fully ginglymous. Both metatarsals bear a pit for the collateral ligament on the medial surface of the trochlea. 

**Figure 6:**
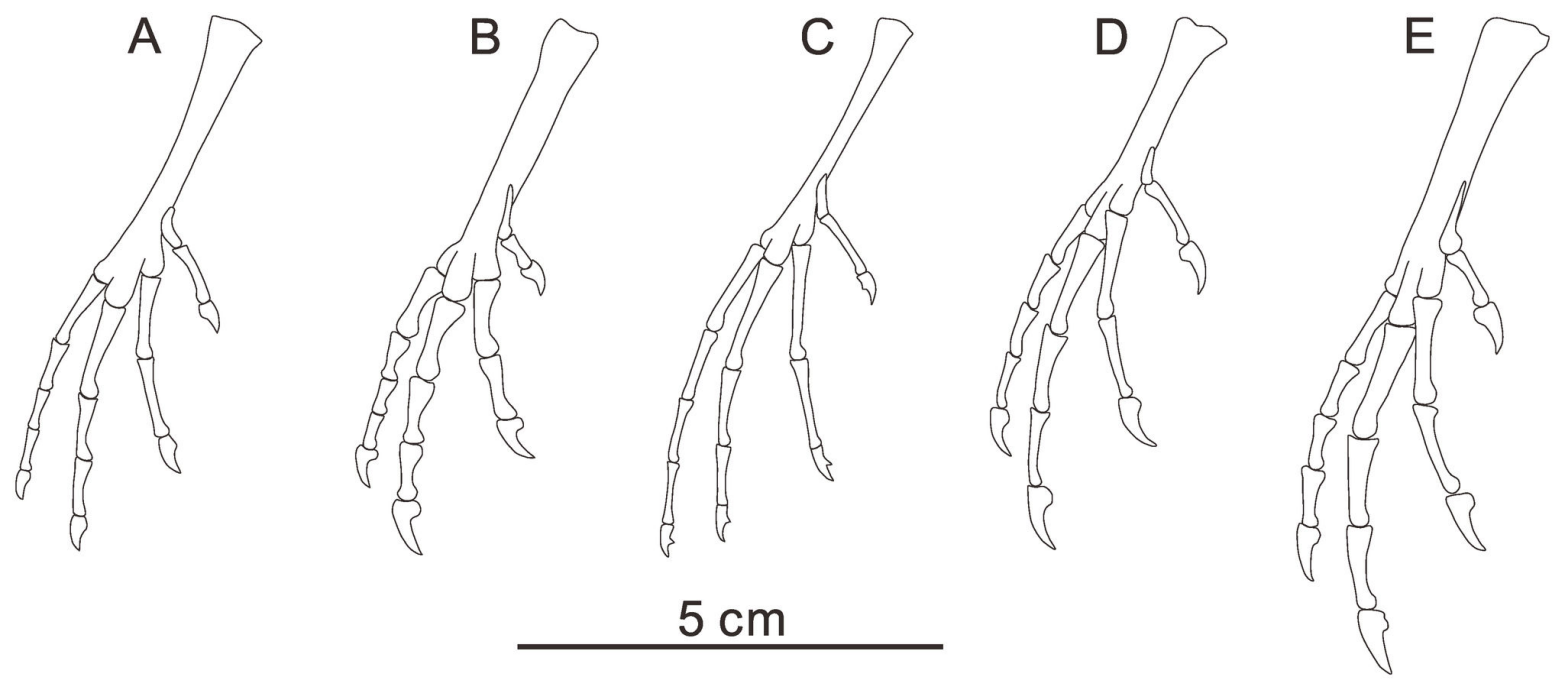
Comparison of pedes of *Changmaornis houi* (A) and other Early Cretaceous ornithuromorphs. B, *Schizooura* *lii*; C, *Gansus yumenensis*; D, *Yixianornis grabaui*; E, *Yanornis martini*.

The pedal phalanges are well preserved (Figure 5). Digit I is completely reversed, as in *Gansus*. The first phalanx in each digit is proportionately shorter than that of *Gansus* (CAGS-IG-04-CM-008 and GSGM-05-CM-014; Table 2). The nonungual phalanges of digits II-IV are also shorter than those in *Gansus* (Table 1; Figure 6C). Digit III is the longest, as in most ornithuromorphs (e.g. *Schizooura*, *Yanornis*, and *Yixianornis*; Figure 6) including neornithines [10,19,20,23,25], although the digit is shorter than the tarsometatarsus; the ratio of the length of digit III (including the ungual) relative to the tarsometatarsus is approximately 0.82, similar to that of *Schizooura* (0.80, IVPP V16861; Figure 6B) [30]. This clearly distinguishes this specimen from *Gansus* (CAGS-IG-04-CM-008), in which digit IV is the longest and the ratio of the length of digit III (including the ungual) to the tarsometatarsus is nearly 1.21. 

**Table 2:** Measurements (mm) of hind limbs of *Gansus yumenensis* (CAGS-IG-04-CM-008, GSGM-05-CM-014) and *Changma houi* gen. et sp. nov. (GSGM-08-CM-002).

Measurement	Specimen		
	CAGS-IG-04-CM-008	GSGM-05-CM-014	GSGM-08-CM-002
Tarsometatarsus length	31.5	40	36.9
Pedal phalanx I-1 length	8.1	7.3	7.4
Pedal phalanx I-2 length	3.7	4.1	3.9
Pedal phalanx II-1 length	13.7	15.1	10
Pedal phalanx II-2 length	10.4	12.9	9.9
Pedal phalanx II-3 length	4.4	4.6	4.8
Pedal phalanx III-1 length	13.6	13.5	11.4
Pedal phalanx III-2 length	8.5	12.2	7.4
Pedal phalanx III-3 length	7.1	9.0	7.3
Pedal phalanx III-4 length	4.3	4.6	4.2
Pedal phalanx IV-1 length	11.5^e^	12.0	8.5
Pedal phalanx IV-2 length	8.3	9.7	6.3
Pedal phalanx IV-3 length	7.5	8.7	4.9
Pedal phalanx IV-4 length	7.2	9.3	4.9
Pedal phalanx IV-5 length	3.5	3.7	3.6

*e* Estimated value.

The unguals are all small but robust (Figure 5); ungual IV is the shortest, and II and III are the longest. Relatively deep extensor grooves (compared to those in *Gansus*) run the entire length of the medial and lateral surfaces of all the unguals, resembling *Yixianornis* (Figure 6D), rather than restricted to the distal portion as in *Gansus* (CAGS-IG-04-CM-008). Flexor tubercles are nearly absent; the proximal ends have plantarly projected, weak and rounded tubercles that can barely be distinguished from the proximoventral corner of the articular cotyla.

*Jiuquanornis niui* gen. et sp. nov. 

urn:lsid:zoobank.org:act:DF96E3B8-4A15-403D-85BC-EE46517858E6

### Holotype

**Figure 7:**
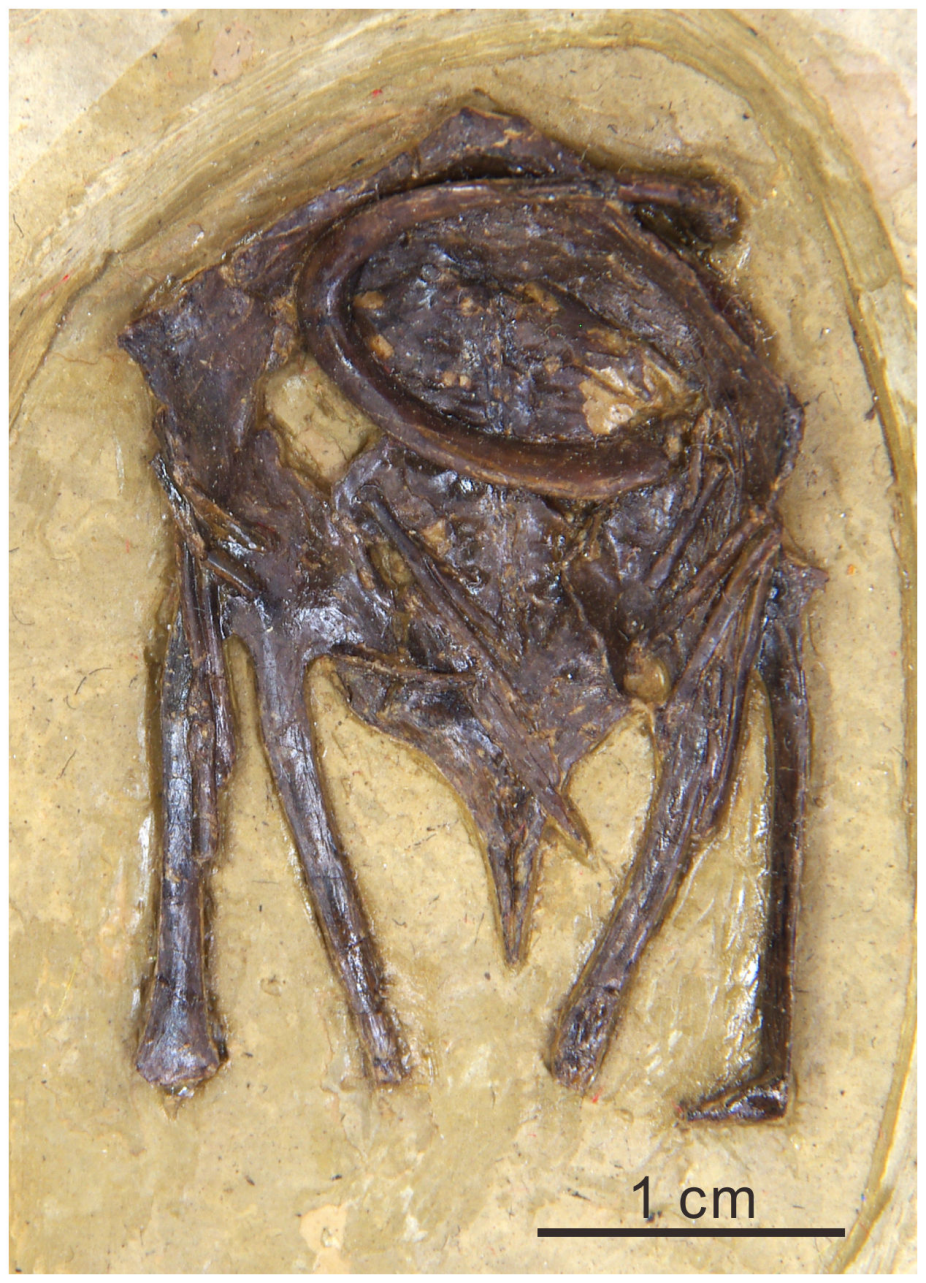
Photograph of *Jiuquanornis niu* gen. et sp. nov., GSGM-05-CM-021.

GSGM-05-CM-021, a complete, associated sternum and furcula, with sternal ribs and possible thoracic rib fragments (Figure 7) [5].

### Etymology

The generic name “Jiuquan” is derived from the name of the city near the fossil locality. The specific name is dedicated to Professor Shao-Wu Niu, for his contribution to geological research in the Changma Basin.

### Diagnosis

*Jiuquanornis niui* is distinguished from other known ornithuromorphs by the following unique combination of characters: U-shaped furcula without a hypocleidium; short imperforate body of sternum; small lateral processes on sternum; lateral trabeculae distally expanded medially; elongate intermediate trabeculae, equal to lateral trabeculae in distal extent; V-shaped xiphoid formed by short, fused medial trabeculae [5]. The sternum of *Jiuquanornis niui* can be clearly differentiated from that of *Gansus* by the long lateral and intermediate caudal trabeculae, V-shaped xiphoid, and absence of caudal fenestrae.

### Comments

*Jiuquanornis niui* was first described as an unnamed ornithuromorph by You et al. [5]. Where comparison allows, the specimen is morphologically distinct from all other described ornithuromorphs, and thus we erect a new taxon. The sternum of *Jiuquanornis* strongly resembles that of *Archaeorhynchus*: both have long lateral and intermediate caudal trabeculae separated by deep incisures, which distinctly separate them from other basal ornithuromorphs; the trabeculae extend well past the caudal midline margin in both taxa. However, the two taxa differ from each other in the following features of the sternum: the rostral margin in *Jiuquanornis* defines an angle of approximately 100°, constituting a sharp craniomedial apex [5], while the rostral margin in *Archaeorhynchus* is more rounded, defining a greater angle of nearly 110°; the craniolateral processes in *Jiuquanornis* are smaller and more pointed than those of *Archaeorhynchus*; *Jiuquanornis* possesses a tapered, V-shaped xiphoid process, while the xiphial region in *Archaeorhynchus* is very short, lacking a distinct process (Figure 3F) [10]. The furcula of *Jiuquanornis* is similar to that of *Archaeorhynchus* and other basal ornithuromorphs (e.g. *Gansus*, *Jianchangornis*, and *Yanornis*); it is U-shaped with a low interclavicular angle of approximately 36°, and lacks a hypocleidium (Figure 7). 

### Phylogenetic Analysis

**Figure 8:**
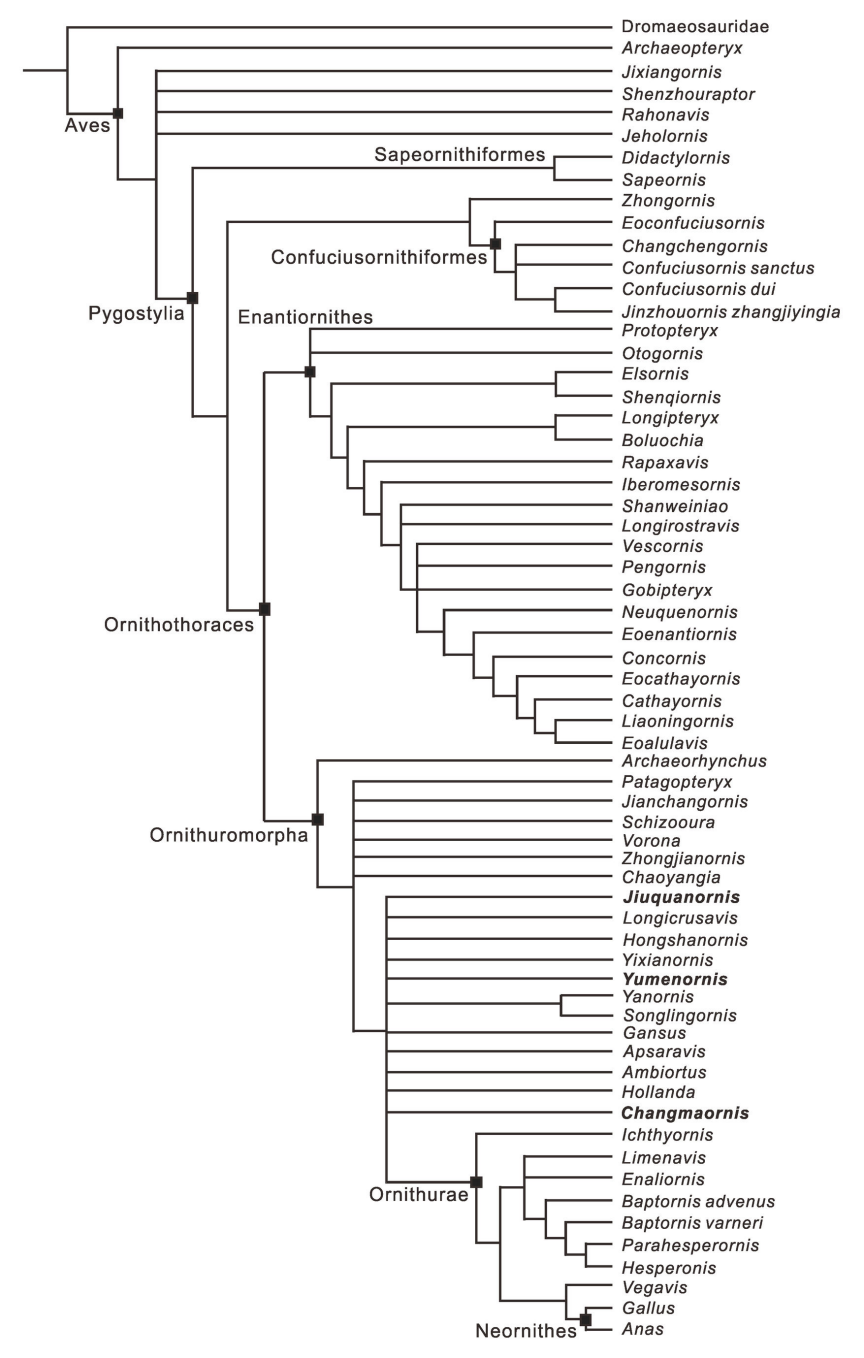
Strict consensus cladogram illustrating the phylogenetic positions of *Yumenornis huangi*, *Changmaornis houi*, and *Jiuquanornis niui*. Tree length: 842 steps, consistency index = 0.387, retention index = 0.670.

The analysis produced 5040 most parsimonious trees (MPTs) of 842 steps. The MPTs differed mainly in the placement of *Zhongjianornis*, *Chaoyangia*, and the three new Changma taxa within Ornithuromorpha. The strict consensus of these trees supports *Yumenornis, Changmaornis*, and *Jiuquanornis* as basal ornithuromorphs (Figure 8).

The relationships among long-tailed birds, basal pygostylians, and enantiornithines concur with the analysis of O’Connor and Zhou [12]. *Archaeorhynchus* is resolved as the basalmost ornithuromorph; *Zhongjianornis*, *Chaoyangia, Patagopteryx*, *Jianchangornis*, *Schizooura*, and *Vorona* form a polytomy with the clade formed by more derived taxa, as in previous analyses [12,18,20,27,31]. However, the addition of the Changma specimens has reduced resolution in the relationships of more derived ornithuromorphs [12,29]: *Yumenornis*, *Changmaornis*, *Jiuquanornis*, *Gansus*, *Yixianornis*, *Hollanda*, *Hongshanornis*, *Apsaravis*, *Longicrusavis*, *Ambiortus*, and *Yanornis* + *Songlingornis* form a large polytomy that includes Ornithurae. 

Although *Yumenornis, Changmaornis*, *and Jiuquanornis* are confirmed to be members of Ornithuromorpha, the interrelationships of these three taxa and their positions relative to other ornithuromorphs remain vague. The phylogenetic hypothesis is weakly supported, as indicated by the low consistency index (0.387) and retention index (0.670); this may result from the lack of data concerning most characters in the largely incomplete new specimens (*Yumenornis*, 81% incomplete; *Changmaornis*, 89% incomplete; *Jiuquanornis*, 94% incomplete; File S1) and the fragmentary or poorly preserved nature of some problem taxa (e.g. *Chaoyangia*, 91% incomplete; *Zhongjianornis*, 63% incomplete) [12]. 

## Discussion

*Yumenornis* is confirmed to be an ornithuromorph bird by the presence of an elongate and imperforate sternal body, a strut-like coracoid with a well-developed procoracoid process, a curved scapula, a prominently convex humeral head, and a craniocaudally expanded first phalanx of manual digit II. *Changmaornis* is also referable to Ornithuromorpha by the presence of a synsacrum composed of at least 11 sacral vertebrae, a completely fused tarsometatarsus, and the plantar displacement of the proximal end of metatarsal III relative to metatarsals II and IV. 

*Yumenornis* can be clearly differentiated from *Gansus* and *Jiuquanornis* based on sternal morphology alone: the rostral margin of the sternum defines an angle of approximately 90°, contrasting with the greater angle in *Gansus*; the lateral process is larger and more rounded than that of *Jiuquanornis*; the sternal body is comparatively much longer than that of *Jiuquanornis*; and the lateral trabecula is short with a large, fan-shaped caudal expansion, contrasting with the long, asymmetrically expanded lateral trabecula in *Jiuquanornis* or the unexpanded strap-like trabecula in *Gansus* (Figure 3). The sternum of *Yumenornis* can be also distinguished from other basal ornithuromorphs by the unique combination of a relatively straight rostral margin with a low intersecting angle (more rounded and with a greater angle in *Archaeorhynchus* and hongshanornthids), a sharp craniolateral process (more obtuse in *Archaeorhynchus*, *Jianchangornis*, *Hongshanornis*, and *Yixianornis*), a large and rounded lateral process (more quadrangular in hongshanornthids and *Songlingornis*; smaller in *Archaeorhynchus*; absent in *Jianchangornis* and *Yanornis*), and a short lateral trabecula with caudal expansion (strap-like in hongshanornthids) (Figure 3). The new specimen is also more robust than previously described Changma ornithuromorphs; the ratio of the width of the midshaft to the total length of the humerus is more than 50% greater than in *Gansus*. *Yixianornis* and *Songlingornis* both possess similar sterna to *Yumenornis*. All three taxa have well developed craniolateral processes, lateral processes, and the short, robust lateral trabeculae all bear large caudal expansions, although there are slight differences in the shape of the expansion compared of *Yumenornis* (potentially preservational). In addition, the length of the forelimb of *Yumenornis* (Table 1) is approximately equal to that of *Yixianornis* (IVPP V13631) [20], and both taxa possess robust humeri (the ratio of the midshaft width to the length of the humerus is 0.1 in both of the taxa) (Figure 4). These similarities may suggest a close relationship between these taxa.

*Changmaornis* can be separated from *Gansus* by several morphological differences: the transverse processes of the synsacrum are longer than those of *Gansus*; the distal half of the pubis in *Changmaornis* is compressed mediolaterally, whereas it is rod-like in *Gansus*; two tubercles on the cranial surface of the distal end of the tibiotarsus contrasting with three tubercles in *Gansus*; pedal digit III is the longest and shorter than the tarsometatarsus (pedal digit IV is longest in *Gansus*, and both digit III and IV are longer than the tarsometatarsus); the grooves on the lateral and medial surfaces of the unguals are deep and run nearly the entire length of the surface (shallow and distally restricted in *Gansus*); and well-developed flexor tubercles are absent (pointed flexor tubercles in *Gansus*). The tarsometatarsus of *Changmaornis* is unique from other basal ornithuromorphs, although it somewhat resembles that of *Gansus*. The metatarsal II trochlea is displaced plantarly and does not overlap with the metatarsal III trochlea in medial view, similar to that of *Gansus*, whereas in other basal ornithuromorphs (e.g. hongshanornthids, *Yixianornis*, *Yanornis*, and *Apsaravis*) the metatarsal II trochlea is only slightly displaced plantarly; in *Changmaornis* this trochlea does not reach the proximal margin of the metatarsal III trochlea, and thus is relatively even shorter than observed in *Gansus* and other Early Cretaceous ornithuromorphs.

*Jiuquanornis* is confirmed to be a member of Ornithuromorpha by its U-shaped furcula lacking a hypocleidium [5]. During the evolution of Aves, the number of fused vertebrae forming the synsacrum increases in more derived taxa [10,12,24]. A synsacrum composed of at least 11 fused vertebrae suggests that *Changmaornis* is a more derived ornithuromorph than *Gansus*, *Yixianornis*, and *Yanornis*. Although unsupported by the phylogenetic analysis, we suggest that *Changmaornis* may also be more derived than *Yumenornis*. The close relationship of *Jiuquanornis* and *Archaeorhynchus* based on numerous similarities of their sterna suggests that *Jiuquanornis* is the most basal member among the four Changma ornithuromorph taxa and potentially is basal within Ornithuromorpha. 

The holotype material of *Jiuquanornis* was originally considered too incomplete to justify the erection of a new taxon [5]; however, most Cretaceous fossil bird specimens from other regions in the world that have been used to erect new species over the past few years are even more fragmentary: the enantiornithine *Bauxitornis mindszentyae* from Hungary is based on a right tarsometatarsus [32]; the enantiornithine *Intiornis inexpectatus* from Argentina is based on a right foot [33]; and the ornithuromorph *Alamitornis minutus* also from Argentina is based on the proximal third of a left humerus [34]. The three specimens from Changma are all more complete than these species and can be distinguished morphologically from other Mesozoic ornithuromorphs, thus we justify erecting a new taxon for each specimen. 

Numerous fossils belonging to Ornithuromorpha and Enantiornithes have been recovered from the Aptian (Lower Cretaceous) Xiagou Formation in the Changma Basin, although no basal, non-ornithothoracine pygostylians have ever been found. *Gansus yumenensis* was the first avian species recovered from the Changma Basin [1], resolved as an advanced non-ornithurine ornithuromorph or basal ornithurine [2,5,10,12,29,35]. With the addition of *Yumenornis*, *Changmaornis*, and *Jiuquanornis*, a total of four named ornithuromorph taxa have been reported now [1,2,5]. 

Enantiornithines also form an important component of the Changma avifauna, with one named taxon, *Qiliania graffini*, and three unnamed taxa [6-9]. Although being less numerous than ornithuromorphs in overall quantity of specimens, enantiornithines formed a greater portion of the taxonomic diversity until the discovery of the three new ornithuromorphs described here [5,6,8,9]. Thus, the hypothesis that the Changma avifauna, which is younger than the Jehol avifauna, may represent an initial stage in the domination of Ornithuromorpha over Enantiornithes both in numbers and taxonomic diversity, is supported here [2,5].

### Body mass in Changma birds

In order to further explore morphological differences between the specimens described here and *Gansus*, as well as between Changma ornithuromorphs and enantiornithines, we estimated body mass for five specimens of *Gansus*, the holotype specimens of *Yumenornis* and *Changmaornis*, and six enantiornithine specimens (including two specimens of *Qiliania*, two reported unnamed specimens, and two undescribed specimens). We estimated the body mass based on Liu et al. [36]; however, since no limb bones are preserved in the holotype of *Jiuquanornis*, this taxon could not be included. The selected specimens of *Gansus* are the most complete and best preserved; however, some of them still lack forelimb or hind limb elements. The humeral length and the tibiotarsal width were measured (Table 3). According to Liu et al. [36], humeral length gives a better estimate of body mass for arboreal birds, while tibiotarsal width is more appropriate for cursorial birds. The width was measured as close to the midpoint as possible in all the measured tibiotarsi. 

**Table 3:** Estimated body masses of fossil birds from Changma.

Species	Humeral length (mm)	Tibiotarsal width (mm)	Estimated weight (g)
Ornithuromorphs			
*Gansus yumenensis*			
CAGS-IG-04-CM-001	?	2.5	?/124.8
CAGS-IG-04-CM-002	?	2.7	?/147.4
CAGS-IG-04-CM-003	48.5	3.0	144.2/185.0
CAGS-IG-04-CM-004	48.0	?	141.7/?
GSGM-05-CM-014	47.8	2.7	140.6/147.4
Mean weight			142.2/142.9
*Yumenornis huangi*	49.9	?	151.5/?
*Changmaornis houi*	?	2.9	?/172.0
Enantiornithines			
*Qiliania graffini*			
GSGM-05-CM-006	?	1.3	?/30.4
GSGM-04-CM-006	?	1.31	?/30.9
Mean weight			?/30.7
CAGS-IG-04-CM-007	?	1.7	?/54.3
CAGS-IG-02-0901	47.7	?	140.1/?
GSGM-05-CM-068	?	1.9	?/69.0
GSGM-05-CM-012	?	2.0	?/77.0

Ornithuromorphs show morphological indicators of a terrestrial lifestyle, while the inferred ecology of basal birds and enantiornithines trends more closely towards arboreal [36-38]. *Gansus* is interpreted as amphibious, potentially a shallow-diving bird [2,4,39], suggesting tibiotarsal width would be a more accurate indicator of body mass. However, the body mass estimates based on each of the two measures are nearly the same, averaging 142.2 g (humeral length) and 142.9 g (tibiotarsal width). *Changmaornis* has a smaller foot than *Gansus*, but potentially it has a slightly greater body mass than most specimens of *Gansus* even though the two ornithuromorphs may have been similar in size. Although lacking hind limb material, *Yumenornis* exhibits a more robust humerus than that of *Gansus*, and it is estimated to have a greater body mass than *Gansus*. *Yumenornis* is estimated to be larger than both *Gansus* and *Changmaornis* (Table 3).

The enantiornithine specimens from Changma that preserve hind limb material all possess long and highly curved pedal unguals, indicative of an arboreal lifestyle [40]. Unfortunately, the absence of preserved forelimb material prevents the most accurate body mass estimates for these specimens [36]. Body mass estimates for Changma enantiornithines are much lower than those for ornithuromorphs (Table 3), consistent with the small and delicate morphology of the preserved material. A similar trend is observed in the Jehol avifauna [36]. 

The proximal position of the metatarsal II trochlea and elongate proximal phalanges of the pedal digits of *Changmaornis* are similar to that of *Gansus*, *Hesperornis*, loons, and grebes [2], thus it is possible that *Changmaornis* was also aquatic. The holotype specimens of *Yumenornis* and *Jiuquanornis* are too incomplete to determine their ecology. However, the potential close relationship between *Yumenornis* and *Yixianornis* suggests *Yumenornis* may have had a more terrestrial lifestyle. The morphology of the sternum in *Jiuquanornis*, with short body and long trabeculae bordering deep incisures, convergently occurs in terrestrial, largely cursorial galliforms and tinamids [5,41]. Only the discovery of more complete materials can clarify these ecological inferences. Comparatively, Changma enantiornithines are considered arboreal. Similar ecological differences are also observed between Enantiornithes and Ornithuromorpha in other regions, especially in The Jehol Group [10,24,36-38,42,43]. Coupled with differences in body mass between these clades, we suggest niche partitioning had strongly developed between Enantiornithes and Ornithuromorpha by the Early Cretaceous. 

## Supporting Information

File S1 **Character states for *Yumenornis huangi*, *Changmaornis houi*, and *Jiuquanornis niui* used in the phylogenetic analysis in this study.**
(DOC)
