# Supplementary material for: Previously Unrecognized Ornithuromorph Bird Diversity in the Early Cretaceous Changma Basin, Gansu Province, Northwestern China
Source: PLoS One. 2013 Oct 11;8(10):e77693. doi: 10.1371/journal.pone.0077693 (PMC3795672; doi:10.1371/journal.pone.0077693)
Supplement: File S1 — Character states for Yumenornis huangi, Changmaornis houi, and Jiuquanornis niui used in the phylogenetic analysis in this study. (DOC) [file pone.0077693.s001.doc]

[Supporting Information](http://www.plosone.org/article/info:doi/10.1371/journal.pone.0063423#s5)

Character states for *Yumenornis huangi*, *Changmaornis houi*, and *Jiuquanornis niui* used in the phylogenetic analysis in this study. See O’Connor and Zhou [12] for character list and states for remaining taxa included in this analysis.

*Yumenornis huangi*

?????????? ?????????? ?????????? ?????????? ?????????? ?????????? ?????????? ?????????? ?11???0?10 0????10000 ??1????2?? ??11011??? 110????010

????????01 ???11??2?? ???3???01? 10000??210 100011???? ??????????

?????????? ?????????? ?????????? ?????????? ?????????? ?????

*Changmaornis houi*

?????????? ?????????? ?????????? ?????????? ?????????? ???????1?1 1??5??????

?????????? ?????????? ?????????? ?????????? ?????????? ?????????? ??????????

?????????? ?????????? ?????????? ?????????? ???????010 ?????0?1?? ??????????

???0?????? 12011???1? ?11?0?20?0 011??

*Jiuquanornis niui*

?????????? ?????????? ?????????? ?????????? ?????????? ?????????? ?????????? ?????????? ?????????? ?????????? ???21002[23]? 221111[123]10? ??????????

?????????? ?????????? ?????????? ?????????? ?????????? ?????????? ??????????

?????????? ?????????? ?????????? ?????????? ?????
